# Personalized Pollen Monitoring and Symptom Scores: A Feasibility Study in Grass Pollen Allergic Patients

**DOI:** 10.3389/falgy.2021.628400

**Published:** 2021-04-08

**Authors:** Letty A. de Weger, Peter Th. W. van Hal, Bernadette Bos, Frank Molster, Marijke Mostert, Pieter S. Hiemstra

**Affiliations:** ^1^Department of Pulmonology, Leiden University Medical Center, Leiden, Netherlands; ^2^Leidse Instrumentmakers School, Leiden, Netherlands; ^3^Leiden Centre of Applied Bioscience, University of Applied Sciences, Leiden, Netherlands

**Keywords:** grass pollen, personal pollen sampler, symptoms scores, pollen-induced rhinoconjunctivitis, allergic rhinitis

## Abstract

**Background:** Pollen is a major trigger for allergic symptoms in sensitized individuals. Airborne pollen is usually monitored by Hirst type pollen samplers located at rooftop level, providing a general overview of the pollen distribution in the larger surroundings. In this feasibility study, grass pollen-sensitized subjects monitored the pollen in their direct environment using a portable pollen sampler (Pollensniffer) and scored their symptoms, to study the relation between symptom severity and personal grass pollen exposure. For comparison the symptoms were also correlated with pollen collected by the rooftop sampler.

**Methods:** After recruitment 18 participants were screened for grass pollen specific (GP-sIgE) of which 12 were eligible. Nine participants completed the study (May, 2018). They were asked to monitor personal pollen exposure using a Pollensniffer on their way to school, work or other destination, and to score their symptoms via a mobile app on a scale from 0 to 10. Daily pollen concentrations were collected by a Hirst type sampler at rooftop level. Pollen grains were analyzed using a microscope.

**Results:** Three of the four participants with high GP-sIgE (≥9.6 kU/l) reported high symptom scores (>4) and an analysis showed a significant correlation (CC) between eye, nose, and lung symptoms and the grass pollen counts collected by the Pollensniffer, as well as the daily grass pollen concentrations monitored by the rooftop sampler (CC≥0.54). In contrast, the participants with low GP-sIgE levels (<9.6 kU/l) reported low symptom scores (≤4) and often other sensitizations were present. For these subjects, no significant positive correlations (CC<0.3) of symptoms with either grass pollen collected by the personal or the rooftop sampler were found.

**Conclusion:** The results of this feasibility study suggest that correlations between the severity of clinical symptoms of grass pollen allergic patients, and grass pollen counts as determined by the Pollensniffer or a rooftop sampler, is restricted to patients with high GP-sIgE levels, high symptom scores, and no relevant other sensitizations. Based on the low numbers of subjects with severe symptoms included in this feasibility study, no conclusions can be drawn on the performance of the Pollensniffer in relating symptoms and pollen exposure in comparison with the rooftop sampler.

**Trial Registration:** The study was approved by the Committee Medical Ethics of the LUMC (approval numbers: NL63953.058.17/ P17.304).

## Introduction

Late spring and summer is the period that 33% of the European allergic population suffers from symptoms of rhinoconjunctivitis due to grass pollen exposure (1). Grasses are present all over the world and grass pollen is one of the most important sources of allergens causing rhinoconjunctivitis symptoms such as rhinorrhoea, blocked or itchy nose, itchy or tearing eyes, and cough. Rhinoconjunctivitis symptoms can be mild but may also have a great impact on the daily life of patients, as demonstrated e.g., by studies in adolescents showing reduced school performance and academic achievements in symptomatic subjects (2, 3). In addition to these known effects, a new rhinoconjunctivitis associated phenomenon appeared in 2020, when it became apparent that some of these symptoms of, like rhinorrhoea, nasal obstruction and cough, were easily misjudged as symptoms of COVID-19, leading to unnecessary anxiety in patients suffering from pollen-induced rhinoconjunctivitis. Informing patients when and which pollen are present in the air will help them better recognizing their symptoms as pollen-induced rhinoconjunctivitis symptoms.

In Europe, a network of more than 500 stations monitor the daily airborne pollen concentrations (4). Information on how many and what type of pollen is in the air is relevant for patients, patient care and research (5). Pollen samplers used for these monitoring purposes are often located on top of buildings at a height of ~20–30 m. Rojo et al. (6) showed that pollen concentrations collected at a height above 10 m are lower and more homogeneous, but still representative for pollen concentrations at near ground. Several recent studies demonstrated the relation between pollen exposure and symptom severity. A close relationship has been demonstrated between symptom scores that are collected among allergic individuals in the general public using mobile applications and pollen levels monitored by stationary samplers located at rooftop (7–9). Damialis et al. showed in an alpine and urban environment that human exposure to reduced natural pollen concentrations resulted in reduced symptoms and immune responses in grass pollen allergic patients (10). Also in cypress allergic patients a significant association between natural exposure to cypress pollen and allergic symptoms was demonstrated, with a plateau effect for the high exposures (11).

In a recent study, we used a mobile pollen sampler to demonstrate that pollen exposure can differ significantly from one location in a city to another (9). These findings underline the notion that allergic subjects will encounter variable pollen concentrations on their way to e.g., school or work, which may explain discrepancies between the pollen measured by the pollen monitoring station and the symptom severity experienced by patients. Therefore, especially personal sampling in the immediate environment of the patient would contribute to understanding symptom development.

Such a personal sampling approach was also found to be useful in other circumstances, as illustrated by the two cases described by Fiorina et al. (12). This study showed that for two allergic patients, who could not clearly be diagnosed by skin prick tests, the responsible allergen was identified by personal sampling in the environment of these patients. The same personal sampler was used in a study of Myszkowska et al. (13) where pollen allergic patients sampled pollen.

Recently, we described a portable sampler, the Pollensniffer (14), that can be conveniently used to monitor pollen at different locations including the immediate environment of a patient. The Pollensniffer was validated by mounting the Pollensniffer on the rain cover of the static Hirst type Pollen sampler on the roof of the Leiden University Medical Center (LUMC) and by comparing the pollen counts in both samplers (14). The Pollensniffer was used to study the variable pollen concentrations at street level in a city (14). In the present study, we aimed to investigate whether pollen grains collected by the Pollensniffer in the immediate environment of the grass pollen allergic individuals are related to their symptoms, and to compare this relationship to that between symptoms and daily pollen concentrations monitored by conventional stationary rooftop samplers.

## Methods

### Participants

**Figure 1:**
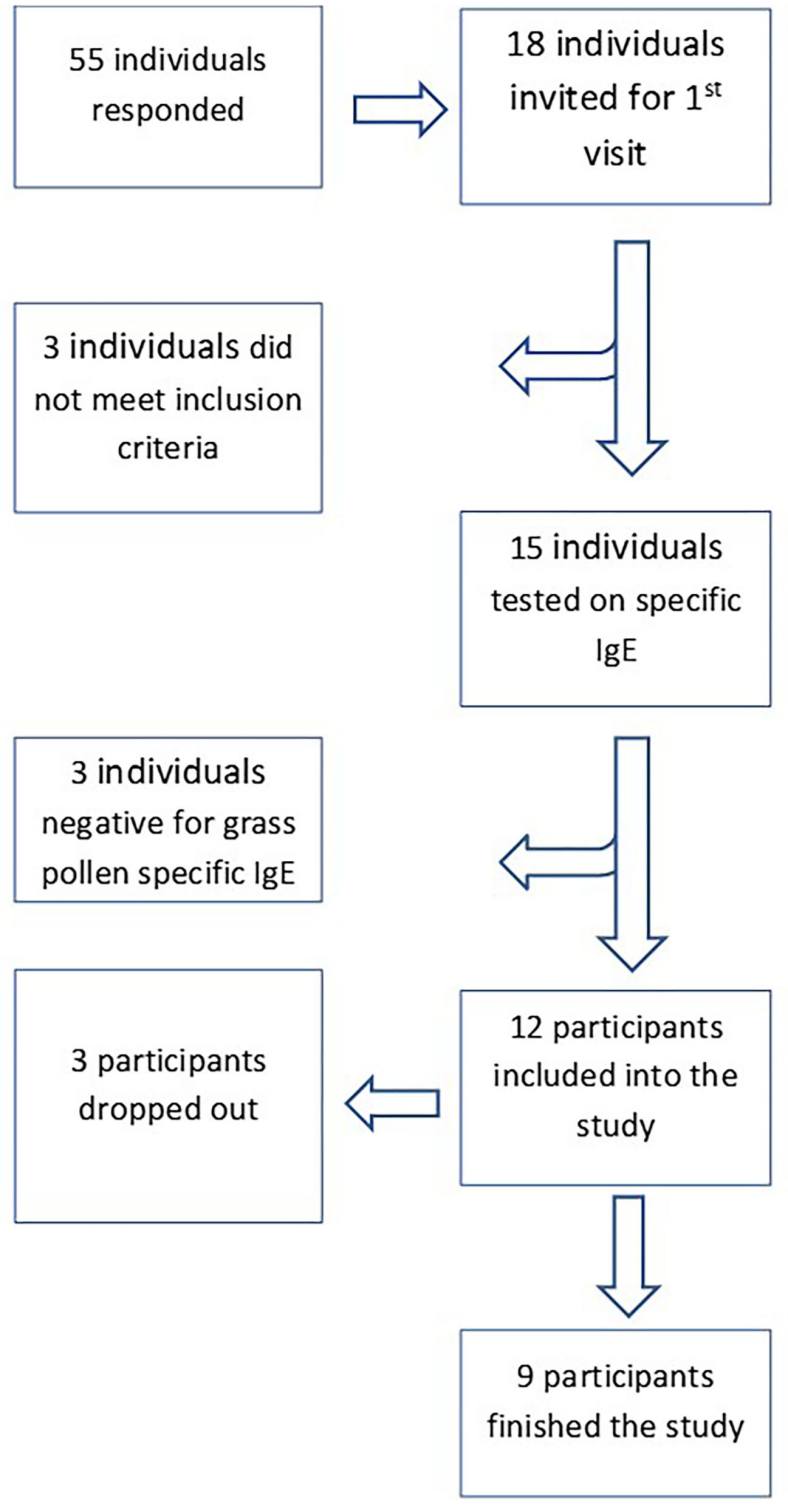
Recruitment and enrolment of study participants.

Participants were recruited in March and April 2018 using social media and posters in the region of Leiden. Fifty-five individuals responded and 18 individuals living in the region of Leiden were invited for a 1st visit (Figure 1). Living within 30 km of the LUMC was relevant to compare the symptom scores with the daily pollen concentrations assessed using a rooftop sampler at the LUMC. Three individuals were excluded due to one or more of the following exclusion criteria: (1) a clinically relevant pet allergy and the very pet at home; (2) immunotherapy, currently or within the last 5 years; (3) daily use of inhaled corticosteroids for asthma; (4) daily use of oral corticosteroids; (5) pregnant or breast feeding; (6) chronically blocked nose; (7) other significant disease (e.g., severe cardiovascular or pulmonary disease, malignancy or autoimmune diseases), where significant is defined as any disease that in the opinion of the investigator would put the safety of the subject at risk by participation. After signing an informed consent, fifteen individuals provided venous blood samples and the serum levels of allergen-specific immunoglobulin E (IgE) were determined by ImmunoCAP (Thermo Scientific, the Netherlands) using a panel of allergens: grass (gx1); birch (t3); mugwort (w6) house dust mite (d1); fungi (mx1); dog (e5); cat (e1). Three individuals appeared to be negative for grass pollen (<0.34 kU/L) and were excluded; the remaining twelve grass pollen IgE positive individuals were included in the study.

The study was approved by the Medical Ethics Committee of the LUMC (approval numbers: NL63953.058.17/ P17.304). All participants provided written consent after receiving a verbal and written explanation of the study.

### Study Design

**Figure 2:**
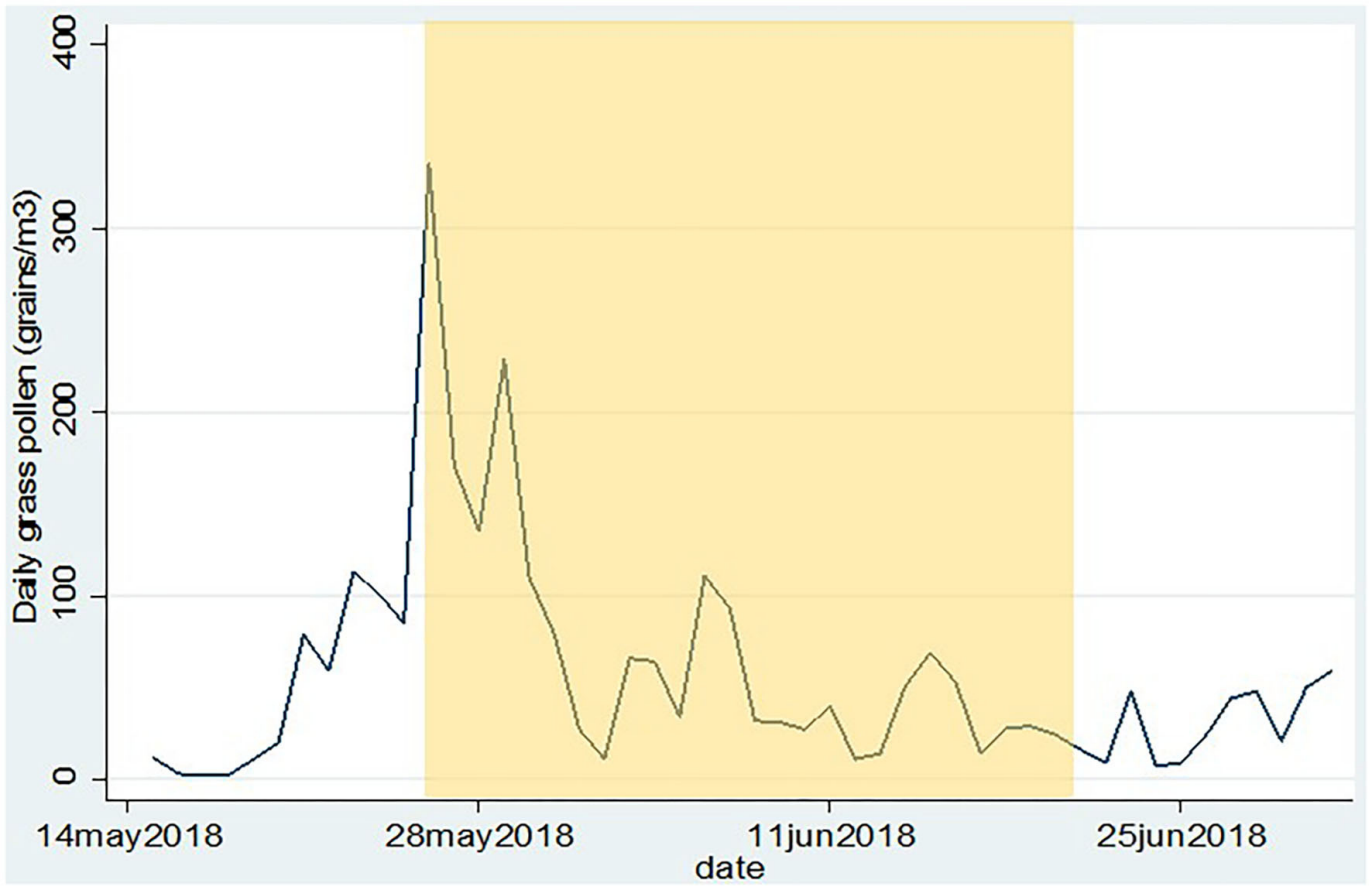
Daily grass pollen concentrations as collected by the Hirst type pollen sampler on the roof of the LUMC. The period in which the participants could collect pollen and score their symptoms is indicated in yellow.

Following inclusion, participants were provided with a subject number (S01–S18) and they received instructions on how to use the mobile application (see later) and the Pollensniffer, either by holding the device in their hand when going on foot, or by mounting the device on their bicycle. The participants were asked to perform the measurements on their first walk or bicycle tour of the day, for instance on their way to work or school during the total time they were outside (exposure time) and to score their symptoms with the mobile application within the next 3 h after collecting pollen. Since the route to school or work varied for each participant, the monitoring time was also different. The sample slides containing the pollen were stored. The participants were asked to collect pollen and score symptoms during 14 days within the next month starting on May 26. They were also asked to write down their mode of transport (cycling or walking) and route. The daily pollen concentrations collected at rooftop level in that period are shown in Figure 2. Participants were asked not to use any medication for their allergic symptoms, starting 3 days before the first measurement. During the study period the participants could contact the clinical research unit for questions or problems. In two cases, the Pollensniffers required small adjustments during use and three participants had issues with the sample slides. After the study period, the participants returned the Pollensniffers and their sample slides and they were asked to fill out a small questionnaire on the use of the Pollensniffer.

### Mobile Phone Application

An application for mobile phones was developed on which users could log in via a personal password, which was linked to their subject number. Users could score their symptoms of eyes, nose and lungs on a scale of 0–10. Upon entering the scores, both location and time were recorded. The data were anonymously stored on a local server.

### Collection of Symptoms- and Pollen Data

Symptom scores were extracted from the server when all symptom scores were submitted (June 20th, 2018). In the Pollensniffer, pollen grains were collected on a Melinex strip covered with Vaseline. The strip was stained with a safranin solution (0.002% w/v) and mounted on a microscopic slide and differential pollen counts were obtained using microscopy (14). All pollen grains, collected on the strip during the walk/ride of the participants, were counted.

For the daily pollen concentrations, the microscopic slides from the Hirst type sampler (rooftop level counts) were scanned using the microscope in three longitudinal bands corresponding to 1 m^3^ collected air in 24 h, to obtain daily concentrations (15).

### Data Analysis

A Shapiro-Wilk test showed that the pollen data from the Pollensniffer or from the Hirst type sampler on the roof were not normally distributed. After log-transformation, the pollen data were normally distributed. Most individual symptoms scores of the patients were normally distributed according to a Shapiro-Wilk test. Pearson correlations coefficients were calculated between the log-transformed pollen data and the individual eye, nose and lung symptom scores of the participants. The grass pollen specific IgE (GP-sIgE) levels of the participants were not normally distributed and thus the geometric mean of the IgE levels was determined instead of the arrhythmic mean.

All statistical analyses were performed using the statistical software package STATA 14.2 (StataCorp, TX, USA).

## Results

### Participant Description

The age of the participants varied between 19 and 56 yr. The group consisted of 3 males and 9 females. The first day of the study period appeared to be a day with very high grass pollen counts (Figure 2) and three participants decided to leave the study since they could not meet the criterium not to use medication; 2 males and 7 females completed the study. The GP-sIgE levels in the serum of these patients varied between 1.4 and 93 kU/L with a geometric mean of 9.6 (Table 1).

**Table 1:** IgE levels of the 12 participants included in the study.

**Patient**	**Grass pollen specific IgE level (kU/L)**						
	**Grass**	**Tree**	**Weed**	**HDM**	**Dog**	**Cat**	**Fungal**
**GP-sIgE level** **>9.6 kU/L**							
S01	93	0	0	0	0	0	0
S06	99	5.4	0.59	0	0.38	0	0
S15	30	0	0	0	0	0	0
S07	10	2.6	0	6.2	0.48	2.8	0
**GP-sIgE level** **<9.6 kU/L**							
S04	7.2	0	0	7.8	0.48	0	0.38
S14	3.8	43	0	0	0	2	0
S03	1.6	1.7	0	0	0	0	0
S16	1.4	1.2	0	5.9	0	0	0
S18	4.3	9.2	0.61	24	0	2.2	0
**Dropped out**							
S09	8.7	3.5	0	28	0	0	0
S13	22	0	0	0	0	0	0
S17	26	0	0	0	0	0	0

*Participants with grass pollen specific IgE (GP-sIgE) levels above and those below the geometric mean of 9.6 kU/L are grouped. The participants that left the study are shown separately*.

### Collection of Pollen

Since the participants were asked to collect the pollen on their way to school or work during the time they were outside, the collection time varied for the different samples in a range from 15 to 40 min. The participants were asked to collect pollen on 14 different days spread over the 4 weeks (May 28th and June 20th, 2018). Most participants (6) collected pollen for 14 days or more, while some (3) participants could only collect pollen during 9, 11, or 13 days. Most samples were collected on a bicycle (Table 2). The range of pollen collected by the participants varied hugely; the lowest number of pollen grains was 2 and the highest number 4,017 pollen grains (Table 2). Although we cannot exclude that the air flows through the Pollensniffer carried by hand while walking or mounted on a bicycle are different, we checked that there was no overall difference in the number of grass pollen collected by cyclists or walkers.

**Table 2:** Characteristics of the data collection.

Range of collection time	15–40 min
No of datasets per person	9–16
Pollen samples collected during	
Walking	44
Biking	74
Biking and walking	2
Unknown	1
Range of pollen collected	2–4,017

Upon return of the Pollensniffer all participants completed a small questionnaire on the use of the Pollensniffer. Most of the participants (6) commented that Pollensniffer was too noisy. Four participants mentioned that the mounting of the Pollensniffer onto the steering wheel of the bike could be improved. Three participants complained about the robustness of the collection box for the sample slides. Participants S03 and S16 had some minor incidents handling 2 and 3 slides, respectively, and participants S04 had an incident with the sampling box which may have affected the integrity of the sample slides. All these slides were microscopically analyzed, and although no discrepancies with the other slides was observed, there may be a chance that the number of pollen grains on the slides was affected by the incidents (see also later).

In all Pollensniffer samples, grass pollen was by far the most numerous pollen type (74% of the total pollen collected by all patients). *Urtica* was the second most abundant species in the total pollen collected (18%). Other allergenic pollen types, like tree pollen, birch, alder, ash or oak, never exceeded 1% of the number of grass pollen. Weed pollen, such as sorrel, plantain or mugwort, never exceeded 5% of the number of grass pollen in the samples.

### Correlation Between Clinical Symptoms and Pollen Collected by Pollensniffer

**Figure 3:**
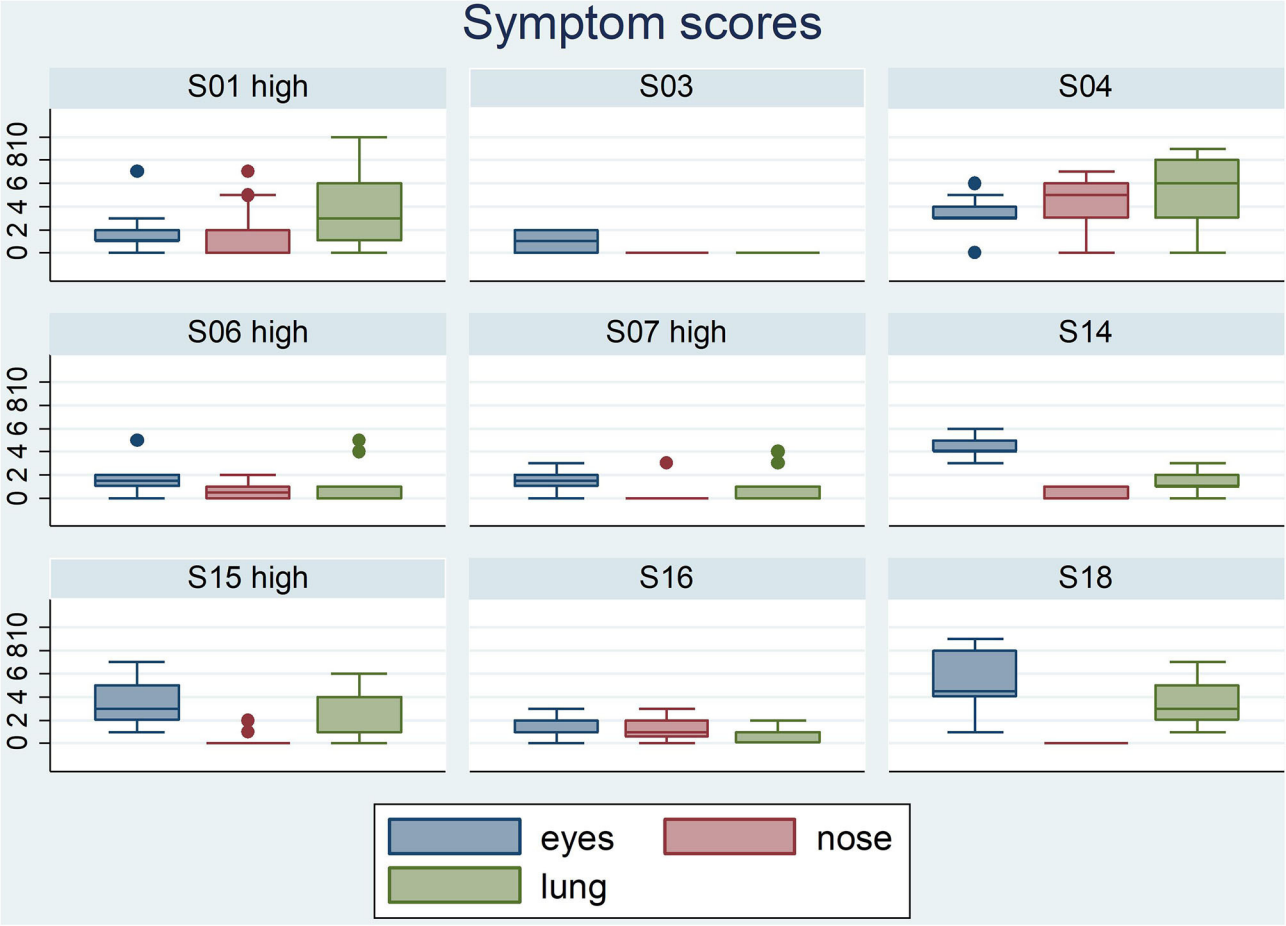
Boxplots of the eyes- nose- and lung symptoms that the 9 participants entered on a scale from 0 to 10. The participants belonging to the group with high GP-sIgE are indicated by the addition “high”.

Participants scored their symptoms on a scale from 0 to 10 using the mobile app. The distribution of the individual symptoms is shown in Figure 3. For some participants the symptoms scores vary only 1–2 scales (e.g., S03 and S16), while others showed a larger variation in symptoms scores (e.g., S01 and S15). In a first analysis, we found significant correlation between either one of the clinical symptom scores and the pollen count in the Pollensniffer samples for only three participants. We noticed that these 3 participants were the ones with the higher GP-sIgE levels and the higher symptom scores. Based on this observation, the participants were split into 2 groups, according to their GP-sIgE levels. Since the IgE levels were not normally distributed we took the geometric mean of the GP-sIgE levels to divide the participants into group 1 (GP-sIgE levels > 9.6 kU/L) and group 2 (≤9.6 kU/L) (Table 1). The different symptoms were correlated with the pollen collected by the participants. Three of the four participants (S01, S06, and S15) from group 1 (high GP-sIgE levels) showed a significant correlation (Supplementary Figure 1A, Table 3) for one (S01 and S15) or two (S06) types of symptoms. These participants had no other sensitizations or other sensitizations with low specific IgE levels (Table 1), and their range of symptom scores was large (from 0 to ≥ 5). The scatter plot of results from participant S07 (Supplementary Figure 1A, Table 3) showed a non-significant moderate correlation for lung symptoms; some data points correlated by increasing symptoms with increasing number of pollen collected, but other data points show a 0-score for the symptoms when relative high numbers of pollen were collected (Supplementary Figure 1A).

**Table 3:** Correlation coefficients between the individual symptoms scores of the 9-grass pollen-sensitized participants and the log-transformed pollen counts in the samples collected by the individuals using the Pollensniffer.

	**S01**	**S06**	**S07**	**S15**	**S03**	**S04**	**S14**	**S16**	**S18**
EYES	−0.145	0.353	0.243	0.654^*^	0.218	0.283	0.277	−0.346	−0.193
NOSE	0.036	0.752^*^	0.141	−0.126	–	0.339	−0.111	−0.207	–
LUNG	0.552^*^	0.705^*^	0.345	0.273	–	−0.326	0.100	0.130	−0.245

* *The green marked cells show significant positive correlations (p < 0.05)*.

The five participants in group 2 (low GP-sIgE levels) showed moderate, none or even negative correlation and between the symptoms and the pollen collected (Supplementary Figure 1B, Table 3). Participant S04 showed a moderate positive correlation. Participants S16 and S03 both had incidents with 2 or 3 slides but leaving out the data points belonging to those slides did not significantly alter the outcome of the analysis. These participants had very low IgE levels to grass pollen and their symptom scores were low (≤2). Participant S14 submitted the symptom scores during the evening and may have scored the symptoms over the whole day and not directly after exposure during the sampling. Participant S18 showed no or negative correlations with all types of symptoms. This participant had relatively high IgE level toward house dust mite and trees. Furthermore, this participant had received grass pollen immunotherapy more than 10 years ago. These conditions might have affected the relation between grass pollen and symptoms.

Since physical exercise may have influenced the symptoms, we studied whether the symptoms collected during cycling differed from when they were collected during walking. Boxplots on the distribution of the eye-, nose-, and lung symptoms collected in these two ways did not indicate significant differences (data not shown). Since the number of data (especially for symptoms collected while walking) was low we did not study this in more depth.

A lag in the symptom development has been described in other studies (10, 16) and we also related the symptoms to the collected log pollen of the previous day (lag-1). Interestingly, participant S15 showed a significant correlation (*p* = 0.635) between the nasal symptoms and the pollen concentration of the previous day (lag_1), whereas the eye symptoms correlated significantly with the pollen collected on the same day (Table 3).

These results show correlations between the severity of symptoms and the personal grass pollen exposure especially in patients with high GP-sIgE levels, high symptom scores and no other relevant sensitisations.

### Correlation Between Clinical Symptoms and Daily Pollen Concentrations at Rooftop Level

We next investigated the relationship between these symptom scores and the pollen counts derived from the nearby local pollen monitoring station collected at rooftop level at the LUMC. Significant positive correlations between symptom scores and daily pollen counts were found in the group with the high GP-sIgE levels (group 1, Table 4). For participants S01 and S15 the same type of symptoms, that correlated significantly with the Pollensniffer pollen counts, showed a significant correlation with the daily rooftop pollen concentrations. Participant S06, that showed significant correlations for lung and nose symptoms with the pollen collected by the Pollensniffer, did not show a significant correlation for any type of symptoms with the daily pollen counts at rooftop level. In contrast, whilst symptoms of participant S07 showed no significant correlation with the Pollensniffer pollen counts, lung symptoms for this participant correlated significantly with the daily rooftop pollen concentrations (Table 4). Two of the participants (S14 and S18) with low GP-sIgE levels even showed a significantly negative correlation with the daily pollen concentrations at rooftop level. These findings illustrate that also when using pollen counts from the rooftop sampler, correlation with symptom scores were especially found in those patients with high symptom scores, high GP-sIgE levels and no other relevant sensitizations.

**Table 4:** Correlation coefficients between the symptoms scores of the 9-grass pollen-sensitized participants and the log-transformed daily grass pollen concentrations at the rooftop of the LUMC by the Hirst type pollen sampler.

	**S01**	**S06**	**S07**	**S15**	**S03**	**S04**	**S14**	**S16**	**S18**
EYES	0.082	0.574	0.384	0.558^*^	0.102	−0.164	0.204	−0.186	−0.346
NOSE	0.044	0.499	0.467	−0.108	–	−0.387	−0.751^*^	−0.263	–
LUNG	0.708^*^	0.377	0.598^*^	0.486	–	−0.451	0.389	−0.082	−0.563^*^

* *The green marked cells show significant positive correlation coefficients (p < 0.05), the pink marked cells show significant negative correlation coefficients (p < 0.05)*.

## Discussion

To our knowledge, this is the first study to correlate personal exposure to outdoor pollen and the severity of allergic rhinoconjunctivitis symptoms in grass pollen allergic participants during several days. In this feasibility study, nine grass pollen allergic participants were enrolled. The participants collected pollen on their first walk or bike tour during the day and the number of collected pollen was related to the symptoms developed after this activity. The time span a participant collected the pollen in the Pollensniffer varied between 15 and 40 min, depending on the way of transport (walking or biking) and the distance to the destination. All pollen collected during this exposure time were analyzed. This set up enabled us to directly correlate the symptoms of the participants to the number of pollen grains, to which they had been exposed.

In this study, the pollen grains were collected by two types of pollen sampler. The small, portable Pollensniffer which collects pollen in the environment of the patient, and the Hirst type stationary pollen sampler on the roof of the LUMC, collecting the pollen produced in the region. In a previous study using the portable Pollensniffer for street level measurements, we showed that pollen counts at a certain time point can significantly differ at various locations in a city (14). This may be one of the reasons why the symptoms of allergic patients living in the same region differ (8). We had expected to find a better correlation between symptoms and the pollen collected with the Pollensniffer in the direct environment of the participant, than between symptoms and the pollen concentrations monitored at rooftop level. However, pollen sampled by either method showed for three out of nine participants a significant relation with the symptoms. These participants had high GP-sIgE levels and often high symptom scores. The relation between high specific IgE levels and symptom severity has also been found in other studies (17, 18). A larger range in symptom scores will result in better correlation with increasing grass pollen concentrations compared to symptom scores that vary only 1 or 2 scales (Figure 3). Furthermore, participants in this high-level GP-sIgE group did not have significant other sensitizations that might have interfered with the symptom development caused by grass pollen. Interestingly, one of the participants (S15) also showed a high correlation between nasal symptoms and the pollen concentrations collected the previous day (lag-1), while the eye symptoms correlated with the pollen concentrations of the same day. A lag phase in symptoms development has also been described previously, and it can manifest for eye or nose as well as for lung symptoms (10, 16).

The five participants with low GP-sIgE often also had sensitizations to other allergens and they showed no or even negative correlations with the Pollensniffer-derived pollen counts or daily pollen concentrations determined at rooftop level. The mild symptoms (S03) or the multiple sensitizations for e.g., house dust mite, tree pollen or cat (S04, S14 S16, and S18) may help to explain the absence of a significant relation with the grass pollen. In line with our findings, Myszkowska et al. also found that only patients sensitized to one single allergen showed a significant correlation between personal sampled pollen and symptoms (13). For the current study it would have been ideal to recruit mono sensitized grass pollen allergic individuals only, but this is difficult. From the 15 persons tested for GP-sIgE only 3 persons were grass pollen mono-sensitized. However, for future studies it is recommended to avoid high co-sensitizations for HDM or trees. Previous exposure to tree pollen or HDM may have primed the patients resulting in a triggering at lower grass pollen exposure levels. Also, the grass pollen immunotherapy of patient S18, given more than 10 years ago, most likely still protected this participant from symptom development upon exposure to grass pollen.

The participants shared their experience with this new device after the study and their comments indicated 3 points of improvement. (1) The noise produced by the ventilator in the Pollensniffer should be reduced (2), the mounting of the Pollensniffer to the steering wheel of the bicycle should be easier, (3) the collection box should be more robust. Especially the weaknesses in the collection box led to incidents with the sample slides without affecting the analysis, but this should be avoided in a future study.

The main outcome of this feasibility study is that focussing on relevant traits of patients is important when studying the relationship between symptom scores and pollen sampled in the patient's environment. To establish such relations, it appeared to be relevant to enroll participants with high levels of GP-sIgE, and thus most likely severe symptoms, and preferentially low levels of sensitizations to other allergens since these may contribute to symptom development independent of grass pollen exposure. In our participants group the number of participants with a high GP-sIgE level was rather low. This was also caused by the fact that three participants with high levels of GP-sIgE levels dropped out of the study, since withdrawing their medication was not possible due to the severity of their symptoms. Since it was an inclusion criterium of the study not to use medication, these participants had to leave the study. Although we aimed to study symptom development without interference of medication, we realize that this prerequisite might have hampered the inclusion of best suitable candidates. For future studies, it is recommended to reconsider this requirement and consider allowing the use of specific medication during the study period; this medication use could be added into the symptom score resulting in a combined symptom-medication score (19, 20), or used as a confounder in the analysis. Furthermore, in this feasibility study we did not include negative control participants e.g., non-allergic individuals. This might be considered in next studies since also non-allergic individuals may show pollen associated nasal symptoms (10, 16).

Allergic individuals may develop late phase nasal allergic symptoms upon contact with pollen depending on the patient's susceptibility and allergen dose (21). Since we asked the participants to send in symptoms within 2 h after pollen collection, we did not consider these late-phase symptoms.

During the analysis of the pollen slides, we noticed a clear difference in size among the grass pollen grains in both the samples of the Pollensniffer as well as in the samples of the rooftop sampler (data not shown). Most likely this reflects the presence of pollen from various grass species in the samples, which may differ in allergenicity and in potential to induce symptoms. Since the routes to work or school are different for each participant, they may collect, not only different amounts of grass pollen, but also different grass pollen species with varying allergenic potential. This could influence the correlation with between the symptoms and the number of grass pollen. However, currently we cannot study this further in detail, since we cannot distinguish the different species in our microscopic analysis. Analysis by Next Generation Sequencing of the different grass species (22, 23) could be used in future studies to relate the symptoms to the number of the different grass pollen species.

In this feasibly study, the number of participants with severe symptoms was too low to draw conclusions regarding the performance of the Pollensniffer in relating symptoms to the personal pollen exposure compared to pollen monitored at roof top level. Our results indicate that it is relevant to select participants with high IgE levels, severe symptoms and no other relevant sensitizations to reveal correlations between personal pollen exposure and symptom development.

## Data Availability Statement

The data can be made available upon request to the corresponding author.

## Ethics Statement

The studies involving human participants were reviewed and approved by Medical Ethical Commitee, Leiden University Medical Center PObox 9600 2300RC Leiden. The patients/participants provided their written informed consent to participate in this study.

## Author Contributions

LW: conceptualization, recruitment of patients, methodology, data management, analysis of data, writing original draft and review and editing of the manuscript. PH: patient screening and review and editing of the manuscript. MM: project management and review and editing of the manuscript. BB: patient recruitment and screening, data management, and review and editing of the manuscript. FM: methodology and review and editing of the manuscript. PSH: conceptualization, supervision, and review and editing of the manuscript. All authors contributed to the article and approved the submitted version.

## Conflict of Interest

The authors declare that the research was conducted in the absence of any commercial or financial relationships that could be construed as a potential conflict of interest.
