# Supplementary material for: Personalized Pollen Monitoring and Symptom Scores: A Feasibility Study in Grass Pollen Allergic Patients
Source: Front Allergy. 2021 Apr 8;2:628400. doi: 10.3389/falgy.2021.628400 (PMC8974794; doi:10.3389/falgy.2021.628400)
Supplement: Supplementary file 1 [file Data_Sheet_1.docx]

Supplementary Figures to

Personalized pollen monitoring and symptom scores: a feasibility study in grass pollen allergic patients

by Letty A. de Weger^1^*, Peter Th. W. van Hal^1,#^, Bernadette Bos^1^, Frank Molster^2^, Marijke Mostert^3^, Pieter S. Hiemstra^1^

Supplementary Figure 1. Scatterplots of the symptom scores and pollen counts (log-transformed) collected by the Pollensniffer. For each participant, only eyes, nose or lung symptoms are shown that showed a significant or the highest correlation with grass pollen counts. Participants with high and low GP-sIgE levels are shown in A and B respectively.

A.

r=0.654

r=0.552

r=0.705

B
